# The use of the connective tissue graft from the palate for vertical soft tissue augmentation during submerged dental implant placement: A case series

**DOI:** 10.1002/cre2.626

**Published:** 2022-07-04

**Authors:** Imantas Vatėnas, Tomas Linkevičius

**Affiliations:** ^1^ Šiaulių Implantologijos Klinika Šiauliai Lithuania; ^2^ Institute of Odontology, Faculty of Medicine Vilnius University Vilnius Lithuania

**Keywords:** autogenous soft‐tissue graft, biologic width, crestal bone loss, graft from the palate, keratinized tissue width (KTW)., soft tissue thickness (STT), thin mucosae tissue, tissue thickening

## Abstract

**Objectives:**

To evaluate the efficacy of the soft tissue augmentation vertically, using connective tissue graft from the palate, during submerged dental implant placement.

**Material and Methods:**

Vertical soft tissue augmentation, using connective tissue graft from the palate, combining with submerged dental implant placement was performed for 50 patients (10 males and 40 females, mean age 57.22 years). Soft tissue thickness vertically was measured in the middle of the alveolar crest with the periodontal probe. After 3 months, healing abutments or multiunits were connected to the dental implants, augmented soft tissue thickness was measured vertically in the middle of the alveolar crest. The vertical soft tissue volume gain was calculated using analysis of variance descriptive analysis, significance set to *p* = .05.

**Results:**

All 50 autogenous connective tissue grafts from the palate healed successfully. The average thickness of the soft tissue grafts from the palate was 1.8 ± 0.41 mm. After 3 months, soft tissue thickness vertically increased from 2.27 ± 0.64 mm to 4.35 ± 0.64 mm. This difference between mean figures, between the groups, before and after soft tissue augmentation was found to be statistically significant *F* (263;477). The mean increase in soft tissue thickness was 2.08 ± 0.71 mm.

**Conclusion:**

It can be concluded that soft tissue augmentation vertically, using connective tissue graft from the palate can be successfully used for vertical soft tissue augmentation.

## 1 INTRODUCTION

The health of peri‐implant tissues is associated with peri‐implant soft tissue thickness (STT) and keratinized tissue width (KTW). Crestal bone loss around dental implant sites will be affected by the STT, if the height of the soft tissue is not sufficient (Suárez‐López Del Amo et al., 2016). Various methods and materials for increasing the physiological thickness of tissues have been described, including connective tissue graft (CTG) for vertical soft tissue augmentation. Puzio et al. declared that the higher soft tissue thickness was, the lower marginal bone loss has occurred (Puzio et al., 2020). Also, a critical value for tissue thickness was determined as no less than 2.88 mm. Ustaoglu et al. reported that titanium‐prepared platelet‐rich fibrin (T‐PRT) could be an alternative for connective tissue graft (Gülbahar et al., 2020). Adequate STT might prevent crestal bone resorption in the osseointegration period. Verardi et al. compared porcine dermal matrix and healing abutment, used as a tenting screw to sustain the soft tissues, for the soft tissue thickening (Verardi et al., 2019). As a result, the use of a healing abutment for, the tenting effect has limited efficacy in obtaining a significant increase in soft tissue thickness (2.25± 0.53 mm). Using a porcine dermal matrix at a time of dental implant placement effectively thickens peri‐implant tissues (3.01 ± 0.58 mm). Wiesner et al. (2010) compared augmented soft tissues with autogenous connective tissue grafts and not augmented tissues at the time of dental implant placement and reported that connective tissue grafts effectively increase soft tissue thickness, thus improving the esthetics (Verardi et al., 2019). Surprisingly, despite 1.3 mm thicker soft tissues in grafted sites, there was no statistically significant difference between groups. However, the authors did not measure the vertical enlargement of peri‐implant soft tissues; in the study, buccal and lingual sites were examined, which represented soft tissue enlargement horizontally. The purpose of this study was to evaluate the efficiency of soft tissue augmentation vertically, using connective tissue graft from the palate, during submerged dental implant placement.

## 2 MATERIAL AND METHODS

### 2.1 Patients

Partially edentulous patients, requiring dental implant treatment in the lower posterior region were recruited for this study from the Oral Implantology clinic of Siauliai, Siauliai, Lithuania. The protocol of this study was approved by Kaunas regional ethical committee for biomedical trials, Kaunas, Lithuania (2020 06 25 No. BE‐2‐52). Patients had to fulfill local inclusion criteria: having no general illness; having no contraindications to the implantation procedure; secondary edentulous in the area of the lower jaw molar and pre‐premolar teeth; bone height ≥ 8mm, measured in volumetric computed tomography; bone width ≥6 mm, measured in volumetric computed tomography; at least 2 mm fixed, keratinized gums are planned at the dental implant site.

The exclusion criteria were:

- suffering from systemic diseases that can affect the healing of wounds in the mouth;
- pregnancy;
- taking medicines that can affect the healing of wounds in the mouth;
- smoking more than 10 cigarettes a day;
- diagnosis of active peripheral periodontitis; and
- poor oral hygiene (plaque index by O'leary >20%).
- Each patient received verbal and written instructions and digitally signed the informed consent form with permission to use obtained data for research purposes before participating in the study.

### 2.2 Soft tissue thickness measurement and augmentation

**Figure 1:**
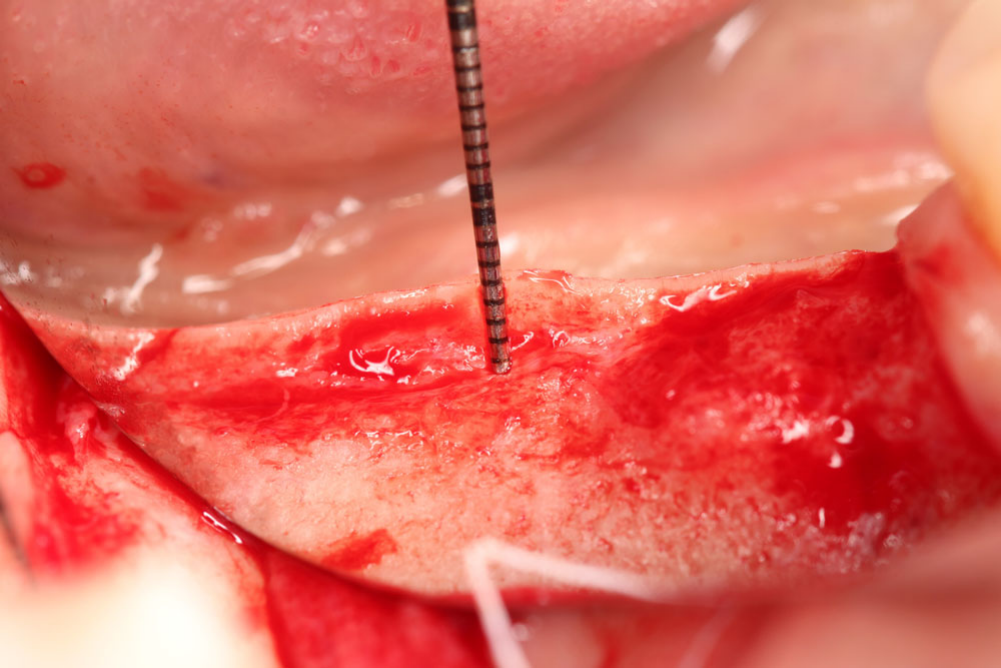
Vertical thickness of soft tissues.

**Figure 2:**
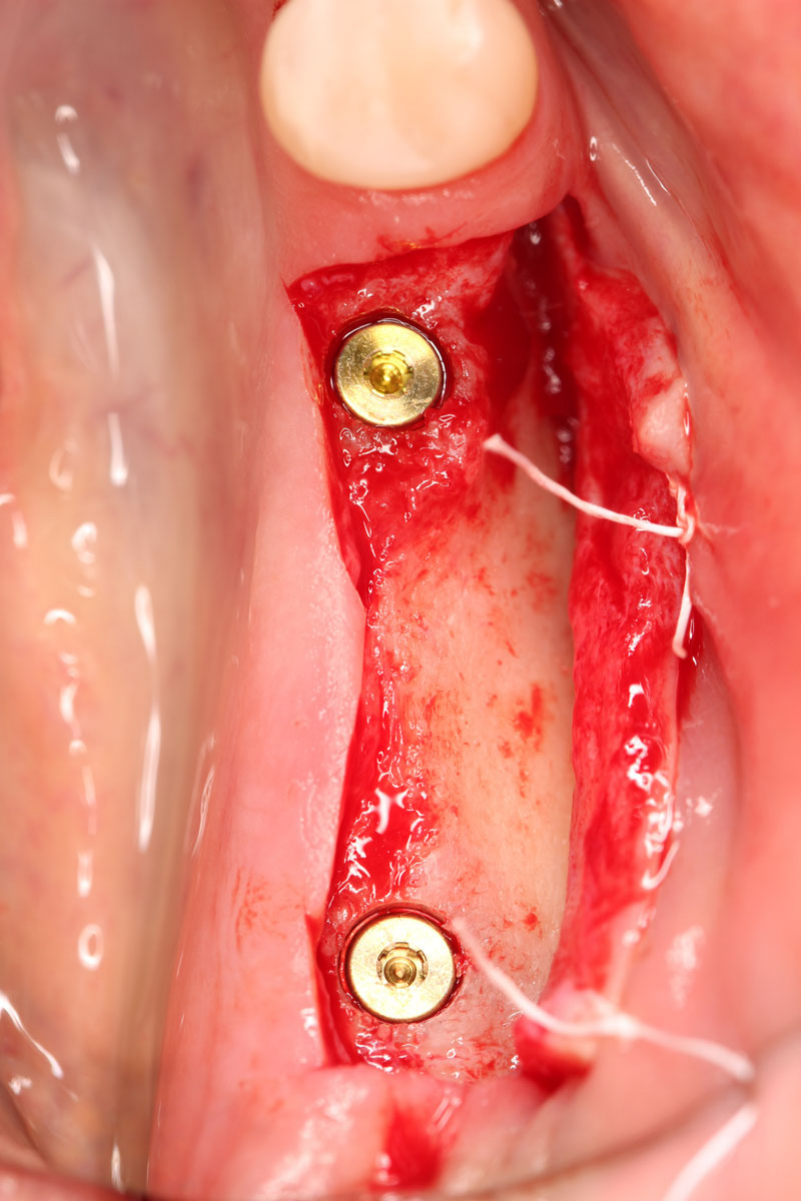
Dental implants were placed.

All surgical procedures were performed by the same surgeon (IV). In the dental implantation site, the width of the keratinized gingiva was measured bucolingually. Surgery was performed under local anesthesia of 4% articaine 40 ml solution with adrenaline (Ubistesin, 3 M ESPE, Seefeld, Germany). An incision with scalpel No.15 in the center of the edentulous ridge was performed. The full‐thickness buccal flap was raised, and the vertical thickness of soft tissues was measured (Figure 1) with a 0.5 mm marked periodontal probe (UNC; Hu‐Friendy, Chicago, IL, USA). The probe was positioned in an upright position to the bone crest in the future dental implant center. Then, a full‐thickness lingual flap was raised to fully expose the dental implant site. The dental implants [“Neodent” (Brazil) dental implant system, Helix, Grand Morse, Acqua 'hydrophilic surface] were placed (Figure 2) according to the recommendations of manufacturers, and dental implant cover screws were connected to the dental implants.

**Figure 3:**
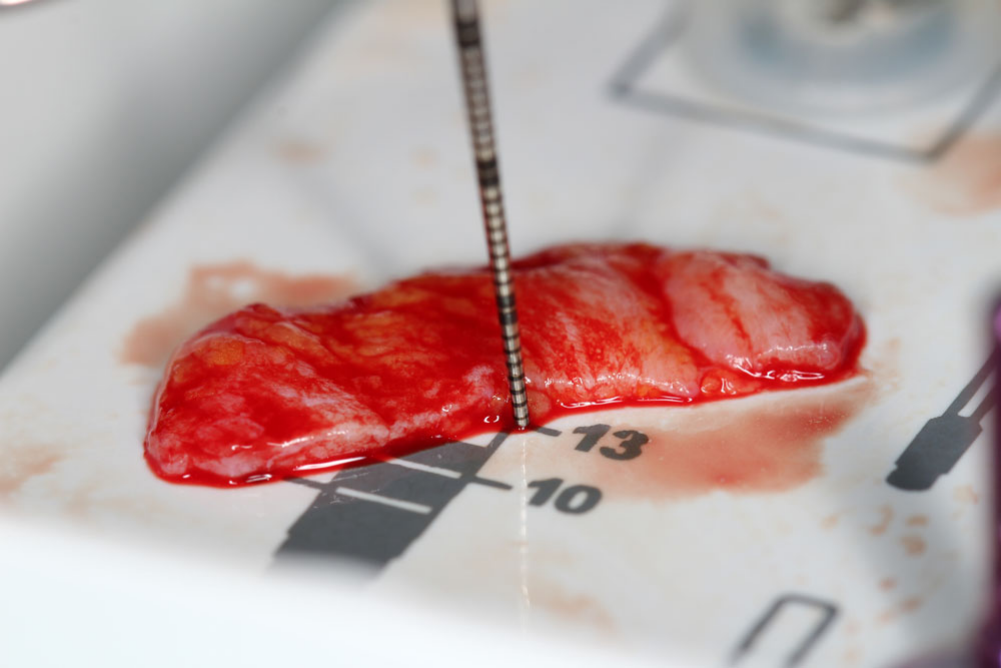
Free gingival graft.

**Figure 4:**
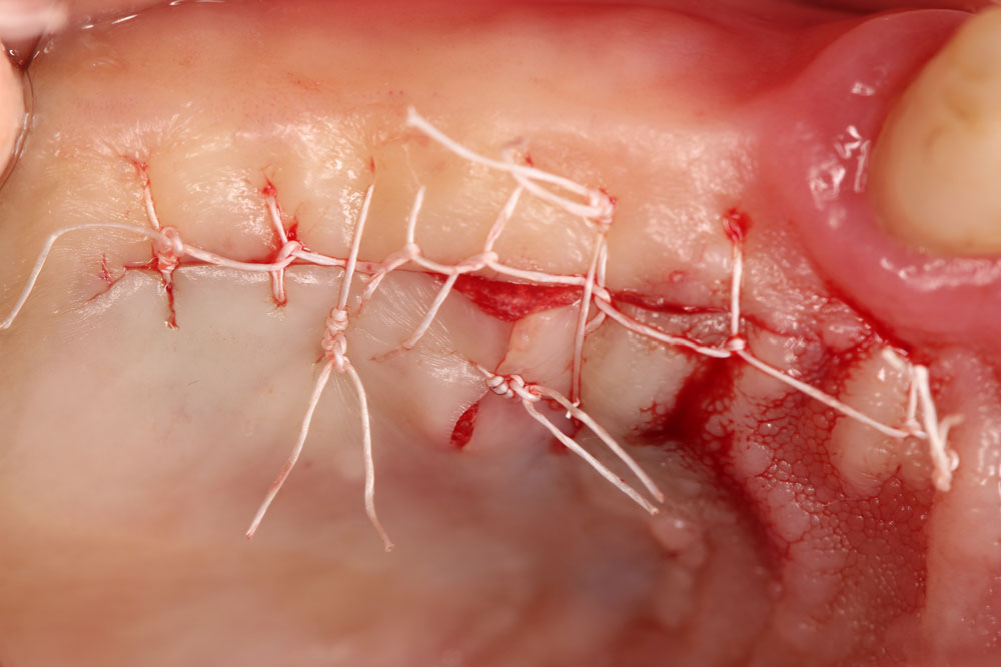
Wound sutured.

**Figure 5:**
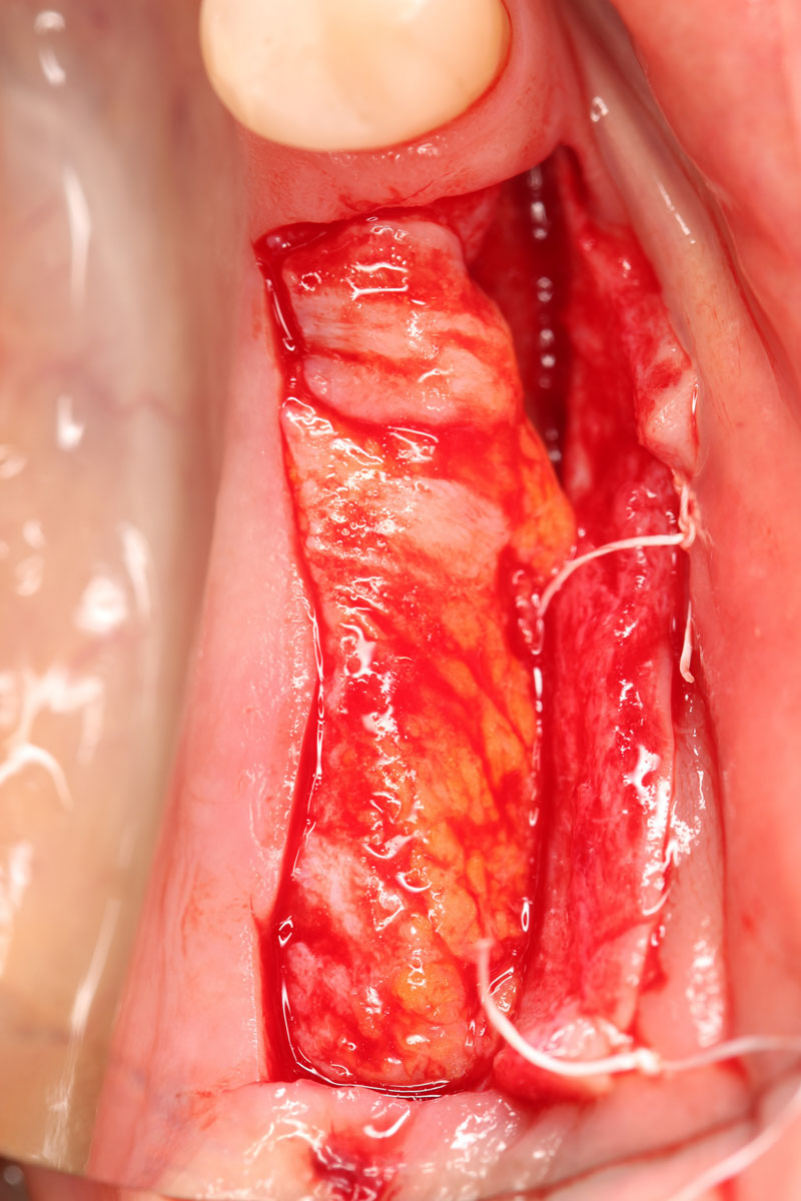
Graft placed over exposed dental implant site.

**Figure 6:**
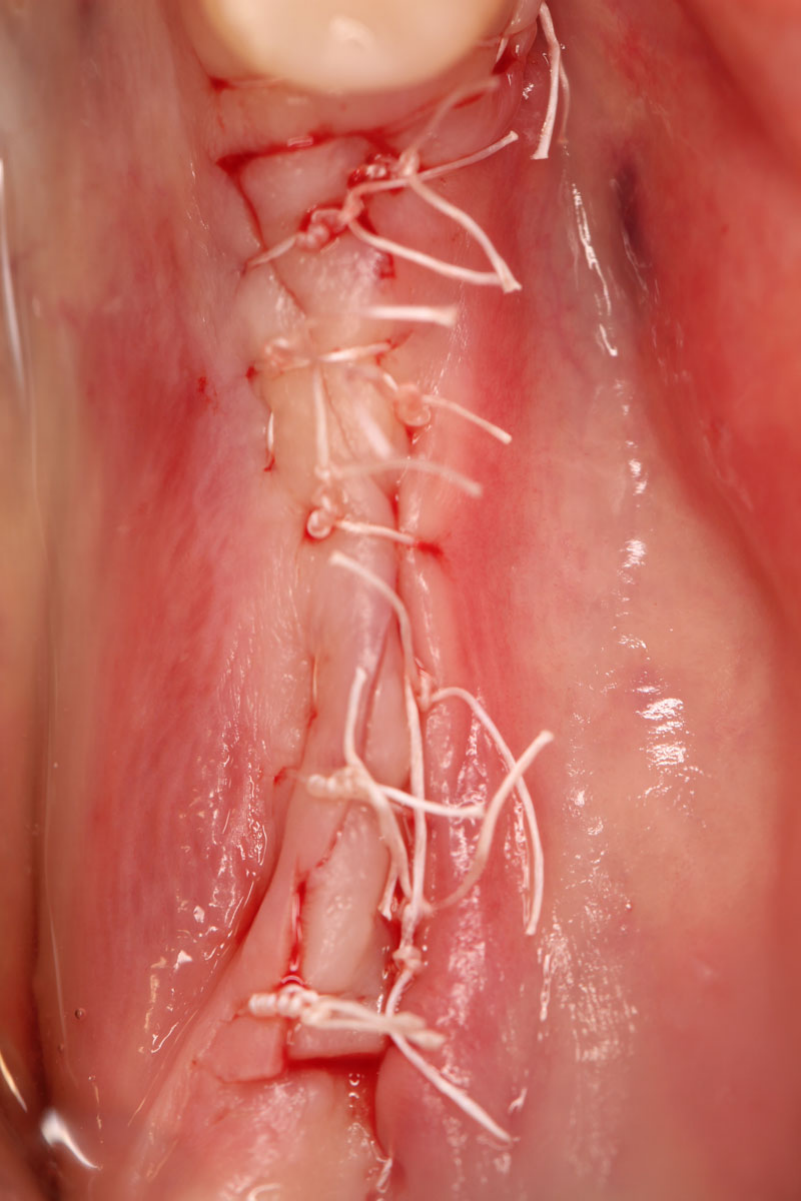
Wound sutured.

After performing local anesthesia (4% articaine 40 ml solution with adrenaline [Ubistesin, 3 M ESPE, Seefeld, Germany]) in the upper jaw in the palate, on the same side, an incision was performed 1‐cm above the gingiva's edge (dental gingival connection), between the first molar and canine. The full‐thickness flap was raised, the flap cut into between, and the inner part taken as a connective tissue graft (Figure 3), wound sutured (Figure 4) without tension with 4/0 sutures (PTFE nonabsorbable monofilament suture “Golnit,” Ukraine). The thickness of the free gingiva graft is measured (Figure 3) with a 0.5 mm marked periodontal probe (UNC; Hu‐Friendy, Chicago, IL, USA) and placed over exposed dental implant site (Figure 5) in the lower jaw, covering the exposed alveolar ridge. Then, coronal periosteal‐releasing incisions were made, flaps were approximated and sutured (Figure 6) without tension with 4/0 sutures (PTFE nonabsorbable monofilament suture “Golnit,” Ukraine). Primary wound closure was always achieved.

**Figure 7:**
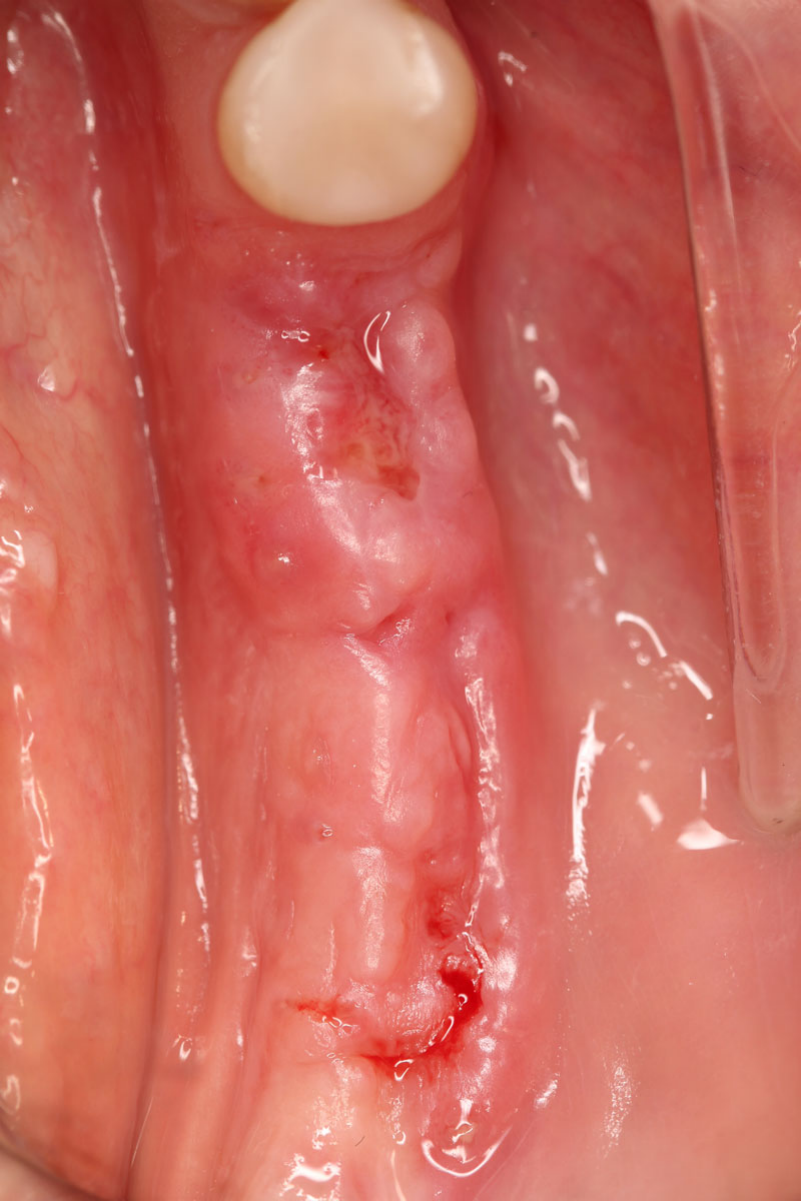
Sutures were removed 14 days after surgery.

After the surgery patients were prescribed antibiotics (1 g amoxicillin [Ospamox; Biochemie, Kiel, Germany]) twice per day for 1 week (or for those with an allergy to penicillin antibiotics, ciprofloxacin, 500 mg twice per daily for 1 week). Pain‐relieving drugs—(400 mg Ibuprofenum [G.L. Pharma GmbH, Lannach, Austria], one tablet twice daily for 5 days). Patients were asked to rise the operated site for 1 min. with 0,12% chlorhexidine digluconate solution (Eludril classic, Boulogne‐ France) three times per day for 2 weeks. Patients were asked to refrain from chewing or brushing the surgical areas for 4 weeks. Sutures were removed 14 days after surgery (Figure 7).

**Figure 8:**
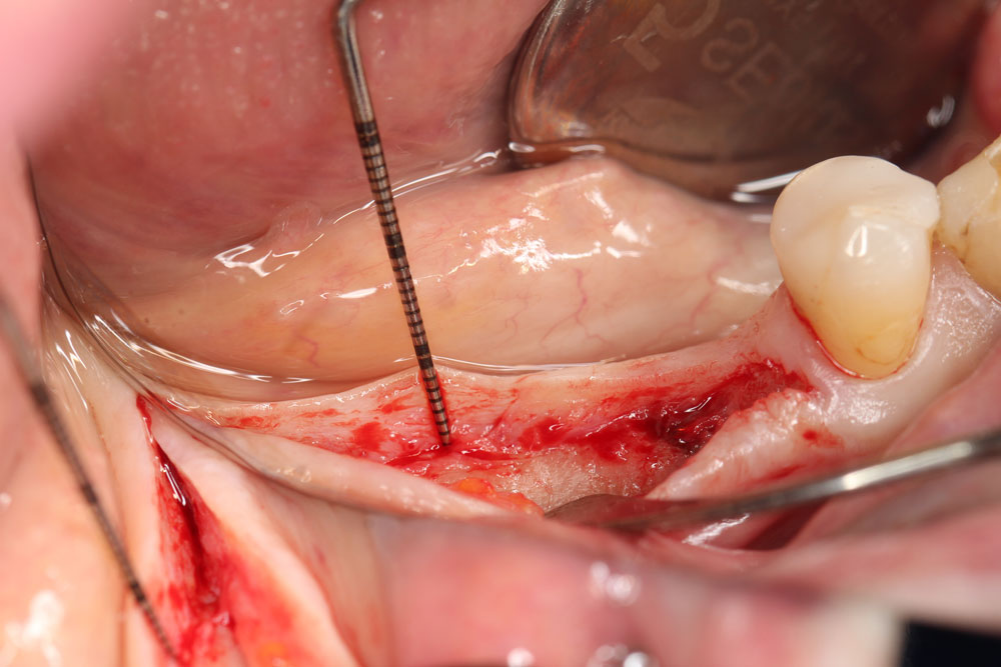
Thickness of augmented soft tissues.

After 3 months of healing, second‐stage surgery was performed. After infiltration of local anesthetic 4% articaine 40 ml solution with adrenaline (Ubistesin, 3 M ESPE, Seefeld, Germany) in the dental implant site, an incision was made in the center of the alveolar crest. A full‐thickness buccal flap was raised, the thickness of augmented soft tissues over the center of the alveolar ridge was measured (Figure 8) with a 0.5 mm periodontal probe (UNC; Hu‐Friendy, Chicago, IL, USA). Then, the lingual flap was raised to fully expose dental implants; cover screws were removed, and healing abutments or multiunits connected to the dental implants. Flaps were approximated and sutured with single interrupted 4/0 sutures (PGLA braided, violet, rapidly absorbable polyglactin 910 suture, coated, “Golnit,” Ukraine). The sutures were removed after 14 days.

### 2.3 Statistical analysis

Data were analyzed using statistical software (SPSS 15.0 for Windows, Chicago, IL, USA). Descriptive statistics were calculated for the measurements as means, standard errors, standard deviations, medians, and range of the measurements. The single patient was treated as a statistical unit. The distribution's normality was tested to apply parametric analysis; one‐way analysis of variance analysis was applied to find differences between soft tissue thickness before and after soft tissue augmentation, using soft tissue grafts from the palate. The mean differences were evaluated using Fisher's “F” criterion; differences were considered statistically significant when *p* ≤ .05. Additionally, 95% confidence intervals were used for demonstrating differences graphically.

## 3 RESULTS

**Figure 9:**
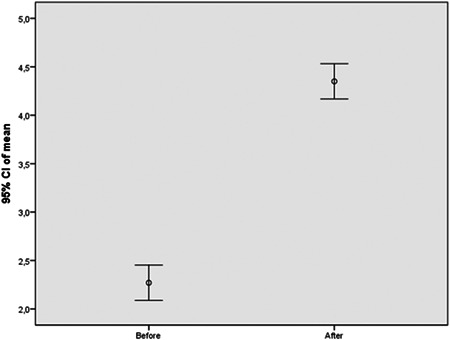
There were differences in measurements before and after soft tissue augmentation procedures in millimeters, and 95% confidence intervals.

Fifty patients were included (average age 57.22 years; range, 38–71 years) at the beginning of the study. Fifty connective tissue grafts from the palate, with a mean thickness of 1.8 ± 0,41 mm were used for soft tissue augmentation vertically in the molar, premolar region, in the lower jaw. All 50 wounds healed uneventfully at the first stage. After 3 months, all grafts showed clinical signs of complete healing: no inflammation, no bleeding. Augmented soft tissues were completely immobile. Soft tissue thickness before the augmentation had an average of 2.27 ± 0,64 mm (Table 1); after soft tissue augmentation, soft tissue thickness increased to 4.35 ± 0,64 mm 3 months after placement. This difference between the mean figures was statistically significant *F*(263;477) (Table 2). The mean increase in soft tissue thickness was 2.08 ± 0.71 mm (Figure 9). Overall, the implant survival rate at the second‐stage surgery was 100%.

**Table 1:** Differences of measurements of (STT) before and after soft tissue augmentation procedures

Descriptive analysis								
					95% confidence interval for mean			
	*N*	Mean	Std. deviation	Std. error	Lower bound	Upper bound	Minimum	Maximum
Before	50	2.270	0.6406	0.0906	2.088	2.452	1.0	4.0
After	50	4.350	0.6409	0.0906	4.168	4.532	3.0	6.0

**Table 2:** Results of one‐way ANOVA testing

ANOVA					
	Sum of squares	df	Mean square	*F*	Sig.
Between groups	108.160	1	108.160	263.477	.000
Within groups	40.230	98	0.411		
**Total**	**148.390**	**99**			

Abbreviations: ANOVA, analysis of variance; df, degrees of freedom.

## 4 DISCUSSION

These case series demonstrate that the soft tissues could be successfully augmented vertically using connective tissue graft from the palate. Using connective tissue graft from the palate‐ simultaneously with the dental implant placement in a two‐stage procedure, soft tissue augmentation vertically resulted in a statistically significant increase of peri‐implant soft tissue height, measured during the second stage of the procedure. The mean increase in soft tissue thickness was 2.08 ± 0.71 mm. As mentioned previously, Wiesner et al. (2010) reported enlarging soft tissues by 1.3 mm with connective tissue grafts from the palate for augmentation. However, the authors did not measure the vertical enlargement of peri‐implant soft tissues; in the study, buccal and lingual sites were examined, which represented soft tissue enlargement horizontally. A similar study by Rojo et al. also evaluated horizontal soft tissue augmentation (Rojo et al., 2018). Thick peri‐implant tissues allow formation of the biological width, minimizing resorption of the crestal bone. During clinical trials, Linkevicius et al. (2009, 2010) demonstrated that thin, soft tissues might predispose significant crestal bone remodeling, while naturally, thick mucosa does not induce bone loss.

Other materials, like porcine‐derived xenograft dermal matrix and allograft acellular dermal matrix are optional for STT procedures. Puisys et al. (2014) demonstrated that thin, soft tissues could be successfully augmented vertically with allograft acellular dermal matrix membrane. Using allograft acellular dermal matrix simultaneously with implant placement in a two‐stage procedure, resulted in statistically significant increase of peri‐implant soft tissue height, measured during connection of the healing abutments. This procedure allowed the transformation of thin soft tissues, with a mean thickness of 1.54 mm, to thick soft tissues with 3.75 mm in thickness on average. Very similar results were obtained by Puisys et al. (2019) using porcine‐derived xenograft dermal matrix for STT vertically, with an average soft tissue thickness increase from 1.65± 0.36 mm to 3.45 ± 0.52 mm.

Another advantage of thin soft tissue thickening is that thick mucosa allows the formation of a better restoration emergence profile, resulting in more esthetic and stable outcome (Belser et al., 2000). The dome technique supports the same principle‐ surgical technique to enhance soft‐tissue margins and emergence profiles around implants placed in the esthetic zone, using connective tissue graft from the palate (Irinakis & Aldahlawi, 2018).

Nevertheless, the human factor remains in all clinical measurements. Also, it is not clear whether the increase of soft tissue thickness would prevail after a longer period. Further studies are needed to clarify the long‐term performance of this soft tissue enlargement and technique on crestal bone stability around dental implants.

## 5 CONCLUSION

Within the limitations of this study, it can be concluded that connective tissue graft form the palate can be successfully used for vertical soft tissue augmentation simultaneously with submerged dental implant placement. Depending on the initial mucosa thickness, an average gain of 2.08 ± 0.71 mm can be expected. Good clinical integration of the graft and complete resemblance to the surrounding healthy mucosal tissues can be expected as early as 3 months postoperatively.

## AUTHOR CONTRIBUTIONS

No author received personal financial contributions from the implant manufacturer.

## CONFLICTS OF INTEREST

The authors declare no conflicts of interest.

## Data Availability

Data are available on request due to privacy/ethical restrictions.
